# Asthma in elderly is characterized by increased sputum neutrophils, lower airway caliber variability and air trapping

**DOI:** 10.1186/s12931-021-01619-w

**Published:** 2021-01-13

**Authors:** F. Schleich, S. Graff, F. Guissard, M. Henket, V. Paulus, R. Louis

**Affiliations:** Respiratory Medicine CHU Sart-Tilman, University of Liege, Sart-Tilman Liege, B35, GIGA I3, Liege, Belgium

**Keywords:** Aging, Asthma, Sputum, Eosinophils, Neutrophils, Air trapping, Airway caliber, Hyperresponsiveness

## Abstract

**Background:**

Elderly asthmatics represent an important group that is often excluded from clinical studies. In this study we wanted to present characteristics of asthmatics older than 70 years old as compared to younger patients.

**Methods:**

We conducted a retrospective analysis on a series of 758 asthmatics subdivided in three groups: lower than 40, between 40 and 70 and older than 70. All the patients who had a successful sputum induction were included in the study.

**Results:**

Older patients had a higher Body Mass Index, had less active smokers and were more often treated with Long Acting anti-Muscarinic Agents. We found a significant increase in sputum neutrophil counts with ageing. There was no significant difference in blood inflammatory cell counts whatever the age group. Forced expiratory volume in one second (FEV_1_) and FEV_1_/FVC values were significantly lower in elderly who had lower bronchial hyperresponsiveness and signs of air trapping. We found a lower occurrence of the allergic component in advanced ages. Asthmatics older than 70 years old had later onset of the disease and a significant longer disease duration.

**Conclusion:**

Our study highlights that asthmatics older than 70 years old have higher bronchial neutrophilic inflammation, a poorer lung function, signs of air trapping and lower airway variability. The role of immunosenescence inducing chronic low-grade inflammation in this asthma subtype remains to be elucidated.

## Background

Bronchial asthma is a chronic inflammatory disease of the airways. Because of improved life expectancy, the proportion of individuals aged upper than 70 years old is growing worldwide. Elderly asthmatics represent an important group that is often excluded from clinical studies. However asthma in the elderly exacerbates as often as in non-elderly asthma and different predictors of exacerbations were recently identified with fixed airway obstruction and chronic rhinosinusitis being predictors in the elderly population while eosinophils was a strong predictor in non-elderly asthmatics [1].

Asthma in older adults is either diagnosed after the age of 70 or have a history of long-standing disease. The prevalence of asthma in the most advanced ages is similar to that of younger ages [2]. Asthma in the elderly is often underdiagnosed or diagnosed as COPD thus leading to improper treatment.

In elderly, comorbidities are more frequently encountered [3] and polypharmacy increases the risk of low adherence and interactions between drugs. Moreover, immunosenescence has been associated with chronic low-grade inflammation called inflammaging with incompletely elucidated underlying mechanisms [4, 5].

There are few data on asthma features in elderly people. Asthma is however not uncommon in subjects aged 70 years old or more with prevalence between 3 and 6% [2]. In this study, we wanted to compare clinical and functional features and bronchial inflammation in young, middle-aged and elderly asthmatics and discuss the potential treatment implications of these observations.

## Material and methods

### Subject characteristics

We conducted a retrospective study on a series of 758 patients with asthma recruited from the University Asthma Clinic of Liege between October 2010 and January 2019 after subdividing the population studied in three groups of age: lower than 40, between 40 and 70 and older than 70. The patients came from routine practice to University Hospital and were recruited by two clinicians involved in asthma. Entry criteria were any patients with asthma aged 18 years old or more who accepted to undergo detailed investigation at the Asthma Clinic. The visits were not parts of an asthma trial. All the patients that had a successful sputum induction were included in the study.

Asthma was diagnosed based on the presence of chronic respiratory symptoms such as cough, breathlessness or dyspnoea together with the demonstration of airflow variability. The latter was defined by airway hyper-responsiveness shown by one or more of the following: increase in Forced Expiratory Volume in 1 s (FEV_1_) of > 12% and 200 ml following inhalation of 400 µg salbutamol or inhaled concentration of methacholine provoking a 20% fall in FEV_1_ of < 16 mg/ml. Methacholine challenge was performed according to a standardised methodology as previously described [6]. Subjects were characterised as atopic if they had at least one positive specific IgE (> 0.35kU/l; Phadia) for at least one common aeroallergen (cat, dog, house dust mites, grass pollen, tree pollen and a mixture of moulds).

### Study design

Patients underwent FeNO measurement at a flow rate of 50 ml/s according to the ERS/ATS recommendations (NIOX, Aerocrine, Sweden). FeNO was first measured and followed by spirometry with bronchodilation, sputum induction and blood sampling. All tests were performed on the same day.

Quality of life was assessed using the self-administered Asthma Quality of Life Questionnaire (AQLQ) [7] and asthma control by the Juniper Asthma Control Questionnaire (ACQ) [8].

Sputum was induced and processed as previously reported [9] and was successful in 78% of the patients encountered in our asthma clinic which is similar to previous report [10]. Cell count were estimated on samples centrifuged (Cytospin) and stained with Diff Quick after counting 500 cells (Dade, Brussels, Belgium).

This study was conducted with the approval of the ethics committee of CHU Liege.

### Statistical analyses

The results were expressed as mean ± SD or mean ± SEM for continuous variables; median and interquartile ranges (IQR) were preferred for skewed distributions. For categorical variables, the number of observations and percentages were given in each category. Comparisons between different subgroups were performed with a Kruskal–Wallis test. The Spearman correlation coefficient was used to measure the association between clinical parameters.

The results were considered to be significant at the 5% critical level (p < 0.05).

## Results

### Demographic characteristics

Older asthmatics were more frequently overweight with a BMI of 27 as compared to 23 kg/m^2^ for asthmatics younger than 40 years old (p < 0.001). They also had lower rates of active smokers (8% versus 20%, p < 0.001) (Table 1). We did not find an increased risk of uncontrolled asthma or exacerbations in our asthmatic patients aged > 70. The lowest asthma quality of life was observed in the middle-aged asthmatics due to higher emotional trigger (score of 4.4 points as compared to 5.2 for asthmatics younger than 40 and to 5.4 for asthmatics older than 70 years old, p = 0.019). Asthmatics older than 70 years old had later onset of the disease (55 years old versus 15 for patients younger than 40 and 35 for patients aged between 40 and 70 years old, p < 0.0001) and a longer disease duration (15 years versus 13 years for patients aged between 40 and 70, p = 0.0013). Focusing on treatment characteristics, we did not find differences in terms of Inhaled Corticosteroids (ICS) treatment and ICS dose, treatment with Long Acting B2 Agonists (LABA) or anti-leukotrienes in the elderly asthmatics as compared to younger patients. However the proportion of patients treated with Long acting antimuscarinic agents (LAMA) was higher in older patients (8% as compared to 0.6% for patients younger than 40, p = 0.001).

**Table 1:** Demographic characteristics of patients according to the age group

	< 40 years	40–70	≥ 70	Global p-value
n	186	468	104	
Gender (M/F)	73/113 (39%)	180/289 (38%)	47/57 (45%)	> 0.05
Weight, kg	69 (58–79)	74 (63–86)***	71 (62–86)^###^	0.0002
Height, cm	168 (161–176)	167 (162–175)	164 (158–170)^#^^	0.000004
BMI, kg/m^2^	22.8 (15–41)	26.4 (16–42)***	27 (18–37)^###^^^^	0.000001
Smokers	20%	23%	8%^###^^^^	
Pack-yr	0 (0–25)	0 (0–45)***	0 (0–50) ^##^	0.000001
ACQ	1.7 (1–2.57)	1.9 (1–3)	1,9 (1–3,86)	> 0.05
AQLQ	4.9 (3.9–5.97)	4.5 (3.5–5.6)*	5 (3.8–5.9)^#^^	0.001
Emotional trigger	5.2 (1–7)	4.4 (1–7)**	5.4 (2–7)^^^^	0.019
Environmental stimulus	4.8 (1–7)	4.5 (1–7)	4.8 (1.3–7)	> 0.05
Symptoms	4.8 (1.2–7)	4.3 (1.2–7)	5 (1.8–7)	0.006
Age of onset	15 (5–25)	35 (15–50)***	55 (32–68)^###^^^^	0.000001
Duration of asthma	11 (1–19)	13 (2–35)***	15 (3–40)^###^	0.0013
ICS	400 (0–1000)	500 (0–1000)	500 (0–1000)	> 0.05
LABA, %	58	63	64	> 0.05
LAMA, %	0,6	5**	8^##^	0.001
Anti-leukotrienes (%)	25	25	33	> 0.05
Anti-IL5 (n)	0	5	1	> 0.05
Exacerbations (n)	0 (0–7)	0 (0–10)	0 (0–6)	> 0.05

*BMI* body mass index, *ACQ* asthma control questionnaire, *AQLQ*: asthma quality of life questionnaire, *ICS* inhaled corticosteroids, dose in beclomethasone equivalent, *LABA *long acting B2 agonists, *LAMA* long acting antimuscarinic agents, *IL5* interleukin 5

Comparison between < 40 and 40–70 (*), comparison between < 40 and ≥ 70 (^#^), comparison between 40 and 70 and ≥ 70 (^^^). P < 0.05 (1 sign); p < 0.01 (2 signs), p < 0.001 (3 signs)

### Functional and inflammatory characteristics

Looking at inflammatory biomarkers, we did not find any significant difference in exhaled nitric oxide levels according to the age subgroup (Table 2). We found a significant increase in sputum neutrophil counts with ageing (37% for patients younger than 40, 48% for patients aged between 40 and 70 and 57% for patients older than 70 years old, p < 0.0001). Sputum eosinophils taken in absolute value were higher in the middle-aged group (35% versus 12% for patients younger than 40, p < 0.05). Sputum macrophages were higher in younger patients. There was no significant difference in blood inflammatory cell counts whatever the age group.

**Table 2:** Functional and inflammatory characteristics of patients according to the age group

	< 40 years	40–70	≥ 70	Global p-value
n	186	468	104	
FeNO, ppb	35(15–70)	27 (14–52)	22 (16–49)	> 0.05
Sputum eosinophils, %	1.4 (0.2–9.5)	2.5 (0.2–14.4)	2.7 (0.2–11.4)	> 0.05
Sputum eosinophils, AV	11.7 (0.32–96)	34.6 (4–175)*	25.3 (0.79–239)	0.0083
Sputum neutrophils, %	37 (15.7–65.4)	48.4 (25–68)***	57 (29–81)^###^^^^	< 0.000001
Sputum neutrophils, AV	538 (213–1554)	721 (283–2292)	1160 (323–3527)^##^^^	0.000004
Sputum macrophages, %	34 (14–53)	21 (11–36)***	15 (7–25)^###^^^^	< 0.000001
Sputum lymphocytes, %	1 (0.2–2)	1 (0..2–2)	1 (0.2–1.8)	> 0.05
Sputum weight	2 (1.35–3.4)	2.32 (1.35–3.9)	2.32 (1.24–3,6)	> 0.05
Blood eosinophils, %	3.2 (1.7–5.8)	2.7 (1.5–4.6)	2.9 (1.9–4.8)	> 0.05
Blood eosinophils, /mm^3^	240 (145–465)	220 (120–390)	270 (143–385)	> 0.05
Blood neutrophils, %	55 (49–63)	57 (49–63)	60 (53–64)	> 0.05
Blood neutrophils, /mm^3^	4180 (3315–5157)	4217 (3570–5550)	4220 (3520–5440)	> 0.05
FEV_1_, %	96 (86–100)	90 (76–104)*	84 (69–97)^###^^	0.000004
Reversibility, %	7.7 (3–15)	8 (2.7–15)	11 (3.4–18)	> 0.05
FVC,%	97 ( 89–108)	97 (82–111)	90 (78–101)	> 0.05
FEV_1_/FVC, post,%	82 (77–86)	77 (70–83)**	76 (69–82)^##^	< 0.000001
TLC, %	96 (89–104)	100 (91–112)	102 (96–114)	> 0.05
FRC, %	101	104	106	> 0.05
RV/TLC, %	29 (24–35)	44 (36–51)***	53 (45–60)^####^^^^^	< 0.000001
DLCO, %	78	76	69^#^^	0.0003
KCO, %	93	90	95	> 0.05
sGaw, kPa/sec	0.84 (0.65–1.1)	0.8 (0.6–1.1)	0.7 (0.5–1)^##^^^	0.019
PC20M mg/ml	2 (0.5–7.2)	3,7 (1.17–15)*	8,8 (1.7–16)^#^	0.021
IgE, kU/l	330 (107–624)	112 (46–324)****	83 (29–380)^####^^^^^	0.001
RAST HDM	2.3 (0.3–25.4)	0.3 (0.1–1)***	0.3 (0.1–0.3)^####^	< 0.000001
RAST birch	0.3 (0–4.4)	0.3 (0.1–0.3)*	0.3 (0.1–0.3)^###^	0.00004
RAST cat	0.7 (0.1–14)	0.3 (0–0.5)*	0.3 (0–0.3)^###^	< 0.000001
RAST dog	0.3 (0.1–2.7)	0.3 (0–0.3)*	0.3 (0–0.3)^###^	< 0.000001
RAST grass pollen	2.8 (0.3–25)	0.3 (0.1–0.5)***	0.3 (0.1–0.3)^###^	< 0.000001
RAST moulds	0.29 (0.1–0.3)	0.1 (0–0.3)	0.3 (0–0.3)	0.00012
CRP, mg/l	1.5 (0.6–3.5)	1.75 (0.8–4)	2.3 (1.1–4.5)	> 0.05
Fibrinogen, g/l	2,9 (2.5–3.2)	3,1 (2.8–3.8)**	3,5 (3.2–4.3)^##^^^	< 0.000001

*FeNO* fraction of exhaled nitric oxide, *AV* absolute value, *FEV*_*1*_ forced expiratory volume in one second, *FVC* forced vital capacity, *TLC*: total lung capacity, *FRC* functional residual capacity, *RV* residual volume, *DLCO* diffusion of CO, *KCO* transfer coefficient, *sGaw* airway compliance, *PC20* concentration of methacholine provoking a 20% fall in FEV_1_, *IgE* immunoglobulin E, *RAST* specific IgE, *HDM* house dust mites, *CRP* C reactive protein

Comparison between < 40 and 40–70 (*), comparison between < 40 and ≥ 70 (^#^), comparison between 40 and 70 and ≥ 70 (^^^). P < 0.05 (1 sign); p < 0.01 (2 signs), p < 0.001 (3 signs)

**Fig. 1:**
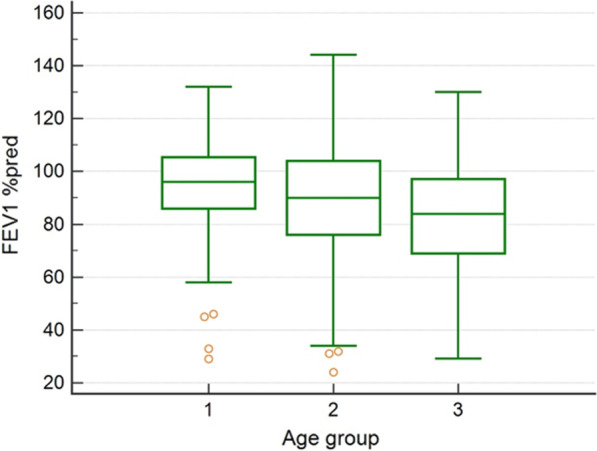
FEV1% predicted value in asthmatics classified according to age. Patients younger than 40 years old (group 1), aged between 40 and 70 (group 2) or older than 70 years old (group 3). Group 1 different from group 2 (p < 0.05) and from group 3 (p < 0.001). Group 2 different from group 3 (p < 0.05)

**Fig. 2:**
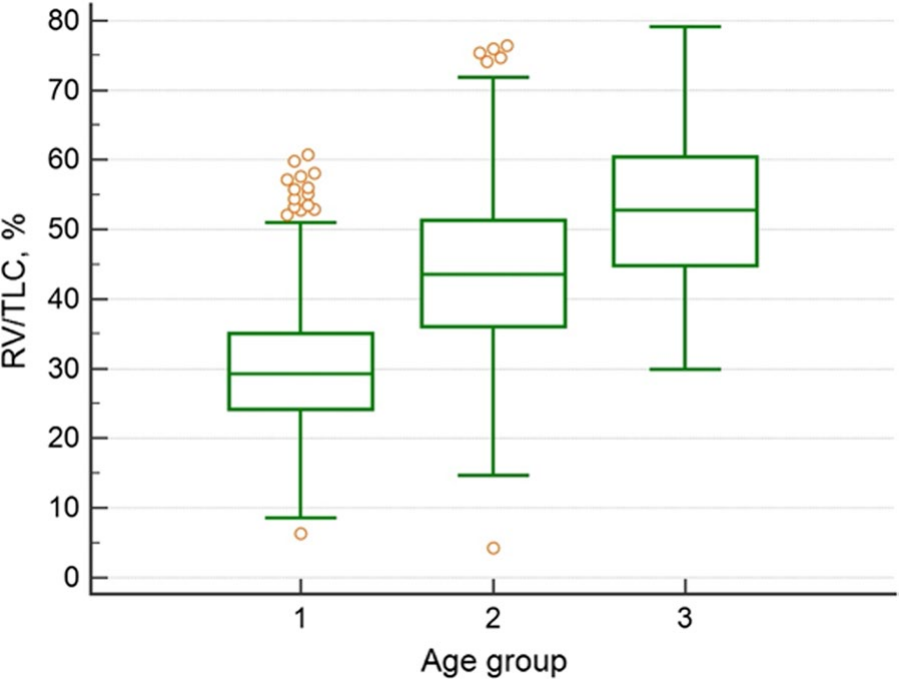
Residual volume on total lung capacity ratio according to age group. Patients younger than 40 years old (group 1), aged between 40 and 70 (group 2) or older than 70 years old (group 3). Group 1 different from group 2 (p < 0.00001) and from group 3 (p < 0.00001). Group 2 different from group 3 (p < 0.00001)

FEV_1_ values were significantly lower in asthmatics older than 70 years old (84% versus 96% for < 40 years and 90% for 40–70 years old, p < 0.05). A same trend was observed for FEV_1_/FVC values that were significantly lower in asthmatics older than 70 years old (76% versus 82% for asthmatics younger than 40, p < 0.001) (Fig. 1, Table 2) while reversibility after bronchodilatation was not different as compared to younger groups. The dose of methacholine required to induce a bronchoconstriction was higher in the elderly asthmatics (8.8 mg/ml versus 2 mg/ml for < 40 years old, p = 0.021). Airway conductance were however significantly lower in asthmatics older than 70 years old (0.7 versus 0.8 kPa/sec for < 70 years old, p < 0.01). We also found signs of air trapping in older asthmatics with RV/TLC of 53% as compared to 29% for asthmatics < 40 years old and to 44% for asthmatics aged between 40 and 70 years old (p < 0.0001) (Fig. 2). Diffusion was found to be significantly lower but KCO was well preserved in elderly asthma.

We found lower levels of IgE (83 kU/l versus 330 kU/l for asthmatics < 40 years old and 112 kU/l for asthmatics aged between 40 and 70 years old, p = 0.001) and a lower rate of sensitization to common aeroallergens in advanced ages (Table 3). Looking at asthma inflammatory phenotypes, we found a higher proportion of neutrophilic asthma (22%, defined as sputum neutrophils > 76%) and mixed granulocytic asthma (10%, defined as sputum neutrophils > 76% and sputum eosinophils > 3%), while there was a lower proportion of paucigranulocytic (33%) and eosinophilic asthma (35%) in older asthmatics (Table 4).

**Table 3:** Proportions of positive skin prick test and/or specific IgE in patients younger or older than 70 years old

	< 70 years (%)	≥ 70 years (%)	p-value
House dust mites	47	21	< 0.0001
Cat	37	9	< 0.0001
Dog	25	8	< 0.0001
Moulds	25	10	0.0007
Grass pollens	39	9	< 0.0001
Birch	20	1	0.029

**Table 4:** Frequence of inflammatory phenotypes between different age subgroups

	< 40	40–70	≥ 70
Paucigranulocytic	N = 91 (49%)	156 (33%)**	34 (33%)^#^
Eosinophilic	70 (38%)	220 (47%)*	37 (35%)^
Neutrophilic	20 (10%)	85 (18%)*	23 (22%)^##^
Mixed granulocytic	5 (3%)	7 (2%)	10 (10%)^#^^^^

Comparison between < 40 and 40–70 (*), comparison between < 40 and > 70 (#), comparison between 40–70 and > 70 (^). P < 0.05 (1 sign); p < 0.01 (2 signs), p < 0.001 (3 signs)

### Link between air trapping and/or sputum neutrophils and functional and inflammatory characteristics

We found a significant correlation between sputum neutrophilic inflammation and air trapping (r = 0.25, p < 0.0001), reflected by RV/TLC ratio and asthma duration (r = 0.08, p = 0.049) while there was a negative association with FEV_1_/FVC (r = − 0.07, p = 0.048), FEV_1_ (r = − 0.15, p < 0.0001) and sGaw (r = − 0.08, p = 0.02) (Table 5).

**Table 5:** Correlations between sputum neutrophil counts in percentage and functional and inflammatory markers

	Spearman’s coefficient of rank correlation (rho)	95% CI for rho	p-value
RV/TLC	0.25	0.17 to 0.30	< 0.0001
FEV_1_/FVC	− 0.07	− 0.13 to − 0.003	0.048
FEV_1_, % pred	− 0.15	− 0.22 to − 0.09	< 0.0001
sGaw	− 0.08	− 0.15 to − 0.01	0.02
Asthma duration	0.08	0.00007 to 0.15	0.049

**Fig. 3:**
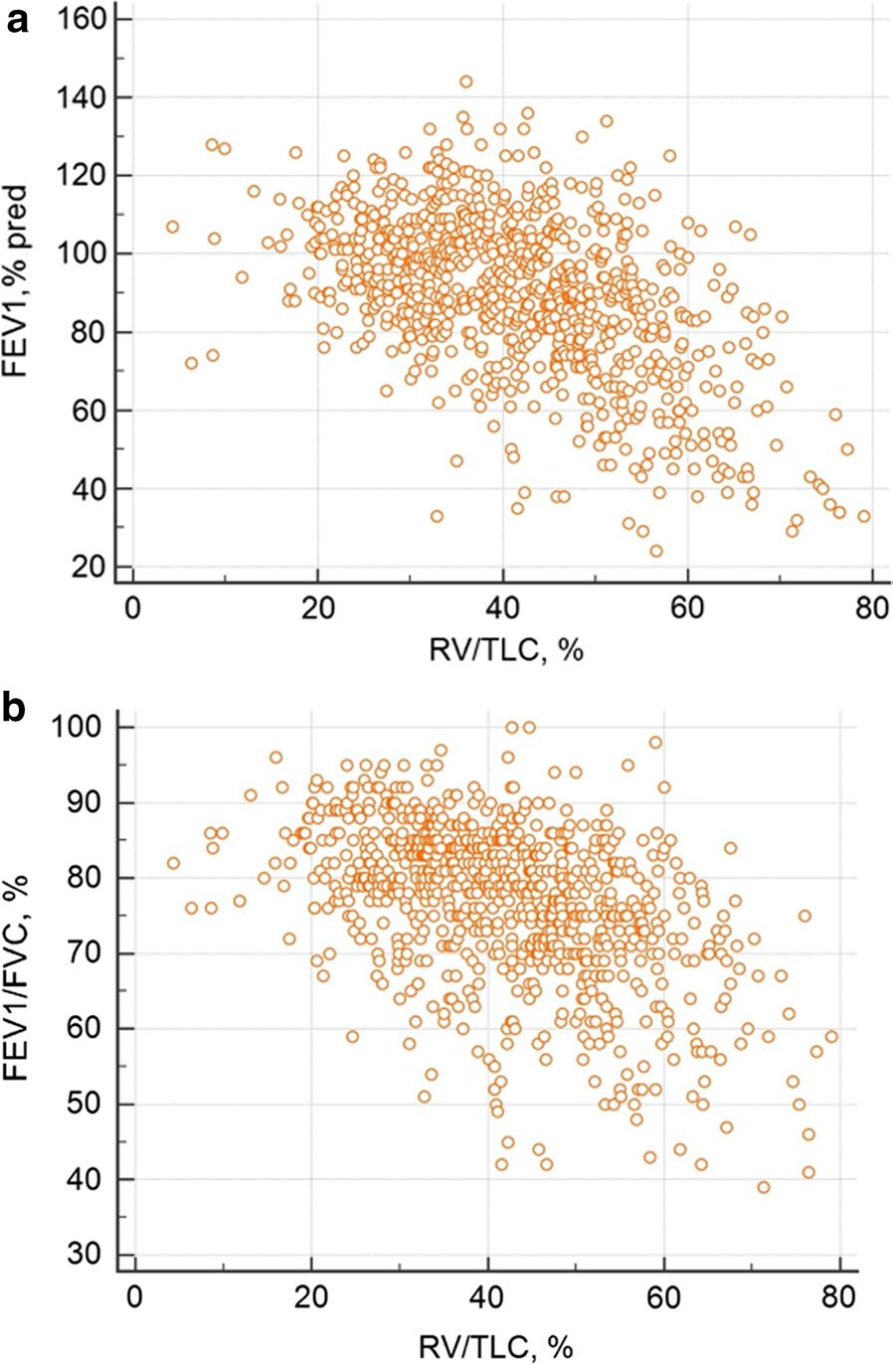
Correlations between FEV_1_ (**a**) and FEV_1_/FVC (**b**) and RV/TLC

Looking at increased residual volume on total lung capacity ratio, we found a significant negative correlation with FEV_1_/FVC (r = − 0.46, p < 0.0001), FEV_1_ (r = − 0.47, p < 0.0001) (Fig. 3) and sGaw (r = − 0.41, p < 0.0001) while there was a positive relationship with asthma duration (r = 0.16, p = 0.0001) (Table 6).

**Table 6:** Correlations between RV/TLC ratio and functional and inflammatory markers

	Spearman’s coefficient of rank correlation (rho)	95% CI for rho	p-value
FEV_1_/FVC	− 0.46	− 0.51 to − 0.41	< 0.0001
FEV_1_, % pred	− 0.47	− 0.53 to − 0.42	< 0.0001
sGaw	− 0.41	− 0.47 to − 0.35	< 0.0001
Asthma duration	0.16	0.08 to 0.23	0.0001

## Discussion

We found that our elderly population of asthmatics was characterized by poorer lung function, limited response to methacholine challenge and signs of air trapping. We showed that older asthmatics exhibit increased sputum neutrophils without any change in blood neutrophils and lower levels of total and specific IgE.

Woodruff et al. previously showed that older age correlated with increased sputum neutrophil percentage but not eosinophil percentage [11]. Although it is well known that neutrophils are increased in elderly [12], it seems that neutrophil chemotactic activity [13] and extracellular DNA traps production [14] declines with age. Our study confirms an increase in sputum neutrophils with ageing with a consequent increase in neutrophilic and mixed granulocytic phenotypes. This increase in sputum neutrophils might not be due to smoking history as there were less smokers in patients older than 70 years old. We found that bronchial neutrophilic inflammation was correlated with air trapping, airway obstruction and asthma duration. We previously found from a multiple logistic regression that age and FRC were independently associated with sputum neutrophilia in a general population of asthmatics [15]. This suggests that airway neutrophils may contribute to reduction of inspiratory capacity seen in some asthmatics. Accordingly, two pediatric studies reported that percentage neutrophils in bronchoalveolar lavage directly correlated with air trapping (FRC) in children with cystic fibrosis [16, 17]. Neutrophils can be retained in the pulmonary microvasculature due to their low deformability, resulting in a higher concentration than in the systemic circulation. It is thought that this high concentration of cells facilitates their effective recruitment to sites of inflammation. It might be that ageing induce tissue remodeling with an increase in microvasculature permeability and air trapping due to a loss of elastic recoil and reduced radial traction in distal airways. The role of immunosenescence inducing chronic neutrophilic inflammation in this asthma subtype remains to be elucidated.

Older age was previously associated with worse lung function [11, 18] and lower bronchial hyperresponsiveness to methacholine. Chuang et al. [19] previously found poorer baseline lung function in the elderly asthmatics while there was no significant difference in percentage of FEV_1_ reversibility between the young and the elderly patients. Our older patients with asthma had a longer disease duration and this could increase the risk of airway remodeling. With regards to this lower lung function, we found a higher proportion of asthmatics treated with long acting anti-muscarinic agents in the older group. Further studies should be performed in elderly asthmatics to evaluate the effect of long acting bronchodilators on airway obstruction in this specific population of asthmatics, often excluded from clinical trials.

We found lower levels of IgE in asthmatics older than 70 years old as compared to younger asthmatics. Moreover, among the six aero-allergens tested, house dust mite was the most common in our population of elderly asthmatics, with one fifth of sensitized patients as compared to half of the younger population. This is line with the results of King et al. [20]. The decrease of prevalence of atopy that we observed in elderly may be explained by a gradual decline in immune function called immunosenescence and changes in tissue structure. The immunoinflammatory responses change during the ageing process as a consequence of continuous damage caused by chronic antigenic stress and repeated environmental aggressions throughout life. As an example it has been found that T cells expressing high levels of CD25 constitutively inhibit the activation of allergen-responsive T cells [21] and increase with age, particularly after 60 years old [22]. As allergen sensitive T-Cells are responsible for B cells activation and the production of IgE, this might explain the observed decreased proportions of patients sensitized to common aero-allergens and the decreased levels of IgE in asthmatics older than 70 years old. Moreover, the elderly have elevated NK cells, most of which are CD8^+^ and produce TH_1_ cytokines with an inhibitory effect on type-2 allergic inflammation [23] and that may induce neutrophilic inflammation. This anti-type-2 or pro-type-1 process is more pronounced in older asthmatics than in healthy elderly.

It was previously suggested that elderly patients were at risk for developing uncontrolled asthma [24]. In elderly, unintentional non adherence with inhalation therapy may lead to significant impairment in asthma control. Complexity of the treatment, cognitive, hearing and visual impairments, low coordination, tremor and arthritis may affect their ability to follow treatment prescribed. Our data show similar results for the asthma control questionnaires in the different age subgroups. Our data are in keeping with the paper of Ponte et al. [18] were age did not predict poor control of asthma if proper treatment was offered.

In a healthy population, FeNO levels evolution throughout ageing were found to reach a plateau between 14 and 45 years in female and between 16 and 59 years in males, then FeNO levels start to increase linearly until 80 [25]. We did not find the same trend in our asthmatic population as FeNO levels were found to be lower in the older subgroup.

In conclusion, our study highlights that asthmatics older than 70 years old have higher bronchial neutrophilic inflammation, a poorer lung function, signs of air trapping and lower airway caliber variability. Immunosenescence and airway remodeling, partly due to longer disease duration certainly play a role in higher airway obstruction, loss of caliber variability and in promoting type-1 inflammation.

## Data Availability

Data are available upon reasonable request.

## Abbreviations

ACQ: Asthma control questionnaire
AQLQ: Asthma quality of life questionnaire
BMI: Body mass index
COPD: Chronic obstructive pulmonary disease
DNA: Deoxyribonucleic acid
FeNO: Fraction of exhaled nitric oxide
FEV_1_: Forced expiratory volume in one second.
FVC: Forced vital capacity.
ICS: Inhaled corticosteroids
IgE: Immunoglobulin E
IQR: Interquartile range
KCO: Transfer coefficient of the lung for carbon monoxide
LAB: Long acting B2 agonists
LAMA: Long acting anti-muscarinic agents
NK: Natural killer cells
SD: Standard deviation
